# Improvements of gamma radiation-induced immunological, hematological, and some biochemical changes in male albino rats by custard apple (*Annona squamosa*) seed oil extract

**DOI:** 10.1186/s12896-025-01062-5

**Published:** 2025-12-01

**Authors:** Amina Aly, Wael El-Desouky, Mahmoud Mohammed, Mohamed AbdEl-Megid

**Affiliations:** 1 Natural Products Research Department, National Center for Radiation Research and Technology, Egyptian Atomic Energy Authority, Cairo, Egypt; 2 Radiation Protection and Safety Department, Hot Laboratories and Waste Management Centre, Egyptian Atomic Energy Authority, Cairo, Egypt; 3 Faculty of Biotechnology, October, University for Modern Sciences and Arts (MSA), Giza, Egypt

**Keywords:** Gamma-rays, Immunological, Hematological, Biochemical, Custard apple

## Abstract

**Background:**

*Annona squamosa* seed oil extract (ASSOE) was used in the current study to investigate the radioprotective potential of ASSOE on radiation-induced hematological, immunological, and biochemical parameters in albino male rats.

**Materials and methods:**

The fatty acid profile of ASSOE was analyzed using GC. Total phenolic content (TPC) and total flavonoid content (TFC) were determined. Antioxidant activity for ASSOE was assayed by the DPPH activity assay, the anticancer activity was also valuated. Forty-nine male albino rats were separated into seven groups: group 1 (normal rats) were administered saline by gavage. Rats in the 2nd, 3rd _,_ and 4th groups were subjected to a single dose of γ-radiation (4, 6, and 8 Gy), respectively, and then administered saline by gavage. The 5th, 6th, and 7th groups of rats were subjected to gamma radiation with 4, 6, and 8 Gy, respectively, then administered ASSOE by gavage (200 mg/kg B.W.) for 14 consecutive days.

**Results:**

*Annona squamosa* seed oil yield was 28.13%, which gave a TPC of 0.71 mg/g ASSOE, TFC of 0.145 mg/g ASSOE and DPPH percentage (65.41%). Fatty acids in ASSOE were oleic acid (57.80%), linoleic acid (23.31%), and palmitic acid (16.22%). The anticancer impact of ASSOE against Caco-2 and HepG-2 cell lines were, 47 ± 0.68 and 912.33 ± 10.81 µg/ml), respectively. Treated rats with ASSOE showed a noticeable increase in the percentage of immune cells, including CD4 and CD8. C-Reactive Protein (CRP) and Tumor Necrosis Factor-α (TNF-α) levels increased following γ-irradiation, but they dramatically dropped after 14 days of administration of ASSOE. Moreover, the 5th, 6th, and 7th groups exhibited notable improvements in all hematological parameters. After 14 days of treatment with ASSOE, cholesterol and triglyceride levels significantly decreased, reversing the rise that occurred following γ-irradiation.

**Conclusion:**

ASSOE is an excellent immunomodulator, radioprotector, and biochemical enhancer, suggesting that it might be used as an adjuvant during radiation therapy.

## Introduction

Radiation therapy, also called radiotherapy, is the application of ionizing radiation to avoid the proliferation of cancer cells and then induce cell death. Despite the radiation risks, radiotherapy has proven to be effective in the treatment of localized cancer in 50% of cancer patients [1]. Exposure of the whole body to ionizing radiation (IR) causes overproduction of reactive oxidative species (ROS) through molecular ionization and, consequently, an increase in cellular oxidative stress. It has been shown that multiple organ dysfunction can be triggered through such effects [2]. On the other hand, ROS can interact with biological macromolecules, such as DNA, proteins, and lipids, and can lead to various pathological manifestations due to alterations in the structure and function of these macromolecules. These inflammation-related changes can result in the development or worsening of cardiovascular disease, atherosclerosis, hypertension, cardiomyopathy, and stroke [3]. Moreover, radiation exposure is notably destructive to the hematopoietic system, causing complications like anemia and leukopenia [4]. Given the potential health risks associated with radiation exposure, particularly for medical personnel, industrial workers, and residents near nuclear sites, there is an urgent need for safe and effective radioprotective agents. Natural products, such as those derived from *Annona squamosa*, represent a potential avenue toward the development of radioprotective agents since they are widely available, economically viable, and have a lower incidence of side effects than manufactured compounds [5]. The search for effective natural radioprotective agents has intensified due to the increased understanding of the detrimental effects of ionizing radiation, which can induce severe alterations in hematological, immunological, and biochemical parameters, leading to substantial health risks [6]. The exploration of natural compounds for potential therapeutic effects has garnered significant attention in both traditional and contemporary medical research. Polyphenols, widely present as secondary plant metabolites, are phenolic compounds possessing multiple hydroxyl groups; based on their structural diversity, they can be classified into numerous subcategories, including but not limited to flavonoids and nonflavonoids, each with distinct physicochemical properties and biological activities. The biological properties of polyphenols, such as anticarcinogenic and anti-inflammatory activities, promote DNA repair, scavenge free radicals, and stimulate hematopoietic recovery and immune functions, which are the main mechanisms of radiation protection [7].

The tropical plant known as the custard apple (*Annona squamosa* L.; *Annonaceae*) is found across South and Central America, Brazil, India, Ecuador, Egypt, and the Bahamas [8]. *Annona squamosa* has been utilized as a naturalistic cure in addition to its several culinary applications. According to Altaee et al. [9], the pulp and seeds of the fruit each had different chemical compositions, including different concentrations of vitamins, minerals, and antioxidants; in addition, each part exhibited a distinct biological activity. Custard apple seeds contain up to 30% oil, yet this byproduct remains underutilized. Recent phytochemical investigations have revealed the presence of bioactive compounds such as acetogenins, flavonoids (quercetin, kaempferol), alkaloids, tannins, and essential fatty acids like oleic and linoleic. These constituents exhibit antioxidant, anti-inflammatory, cytotoxic, and insecticidal activities, positioning the seed oil as a promising candidate for applications in nutraceuticals, pharmaceuticals, cosmetics, and eco-friendly pesticides [10].

Additionally, seed oils of edible fruits are important common food ingredients, and evidence has suggested that some compounds available in seeds may play different roles in human health, especially in acute and chronic disease management, such as cancer, cardiovascular diseases, and diabetes [11]. *Annona squamosa* seeds contain a substantial amount of oil (20–42%), which may be treated with solvent to eliminate the toxic alkaloids and then utilized as detergent or cooking oil [12]. The ASSOE contains significant levels of total tocopherol, associated antioxidant property of phenolic components, and un-saturated fatty acids, particularly oleic along with linoleic acids. In a previous study, the oil’s tocopherol content was shown to be concurrent with the fatty acids; oleic, linolenic, palmitic, and stearic [13].

T-cells come in three primary varieties: cytotoxic, regulatory, and helper. The T-cell receptor is an Ag-binding, Ig-like surface receptor found on the majority of mature T-cells, which express either CD4 or CD8. One subset of lymphocytes is CD4 cells. They play a vital role in the immunological system, their concentrations are a crucial indicator of the health of the immune system. The CD4 cells (helper cells), O methylarmepavine, and Linuginosine (+)-anomuricinem, which might alter the immunological response, are found in *Annona squamosa* bark. The quantity of CD4 + cells, which lead the fight against infections. While the CD8 cells (killer cells) eliminate virus-infected and cancerous cells [14]. Isocorydine, N-methyl-6,7-dimethoxyisoquinolone, linuginosine (+)-CD8+, and CD19 + cells are upregulated, macrophages are activated, T and B cell propagation is promoted, and IL-2 and IFN-γ production is boosted [15]. Some cell types produce TNF-α, including T lymphocytes, Kupffer cells, natural killer cells, and other hepatic cell types. It plays a crucial role in the regulation of inflammatory effects and host defense against microbial pathogens. As a circulatory mediator of innate immunity, TNF-α can cause hemorrhagic necrosis [16].

Although the anticancer and antioxidant properties of *Annona squamosa* seed oil have been demonstrated in few studies, the number of studies investigating ASSOE for its effect in preventing IR-induced damage caused by gamma irradiation is quite limited. In this study, it was aimed to evaluate the expected beneficial effect of *A. squamosa* seed oil in the prevention of the adverse effect caused by gamma irradiation on the hematological, immunological, and biochemical parameters in male albino rats.

## Materials and methods

### Chemicals and reagents

Chemicals for GC standards grade (purity > 95%) were acquired from Sigma-Aldrich (St. Louis, MO, USA) and included ethanol, methanol, petroleum ether (60–80), aluminum chloride, Folin-Ciocalteu reagent, 2,2-diphenyl-1-picrylhydrazyl (DPPH), sodium carbonate, gallic acid, quercetin, fatty acid standard, formazan, and dimethyl suphoxide.

### Plant material: *Annona squamosa*

The current investigation was conducted on *A. squamosa* fruits of the Abd El-Razik cultivar, which were obtained from the local marketplace in Giza Market, Giza, Egypt. The fruit was recognized and validated by Prof. Omaima M. Hafez at the Pomology Department, National Research Centre, 33 El Bohouth St., Dokki, Giza, Egypt.

### Seed oil extract

Fruit seeds were separated from fruits, washed thoroughly using double-purified water, and then dehydrated to a stable weight at ambient temperature (25^°^C). The dried seeds were crushed with a clean coffee grinder, then extracted with petroleum ether (60–80^°^C) using the Soxhlet extraction system at 80^°^C for 6 h. The extracted fixed oil was then kept in brown glass bottles at -20^°^C for subsequent use. The following equation was used to determine the oil yield extract:

$${\rm{Oil}}\,{\rm{Yield}}\,{\rm{(\%) = }}{{{\rm{weight}}\,{\rm{of}}\,{\rm{extracted}}\,{\rm{seeds}}\,{\rm{oil}}} \over {{\rm{weight}}\,{\rm{of}}\,{\rm{seeds}}\,{\rm{sample}}}} \times {\rm{100}}$$

### Total phenolic content

Gallic acid was used as a standard curve to assess the total phenolic content of ASSOE using the Folin-Ciocalteu technique, which is based on Singleton et al. (1999) [17]. The absorbance’s values were taken via spectrophotometer (Jasco-V-530, Tokyo, Japan) at 725 nm. The results were shown as gallic acid milligrams per gram of oil.

### Total flavonoid content

A colorimetric assay of aluminum chloride procedure was employed for determining the total flavonoids of ASSOE, as described by Marinova et al. (2005) [18]. Quercetin was used as a reference; absorbance was measured using a spectrophotometer at 510 nm against a blank (JascoV-530, Tokyo, Japan). Total flavonoid content has been presented as mg quercetin equivalent/gram seed oil.

### Antioxidant activity (DPPH Assay)

The antioxidant activity of the samples was assessed using the technique with respect to the radical 2,2-diphenyl-1 picrylhydrazyl (DPPH) [19].

### Cytotoxicity of *Annona squamosa* seed oil

Colon cancer cell colorectal adenocarcinoma (Caco-2) and liver hepatocellular carcinoma (HepG-2) cell lines were obtained from VACSERA (Giza, Egypt). The modified colorimetric MTT assay is based on the selective ability of living cells to reduce the yellow salt of MTT (3-[4,5-dimethylthiazol-2-yl]-2,5 diphenyl tetrazolium bromide) to formazan, was used to determine the anticancer activity of ASSOE, as reported previously by Van de Loosdrecht et al. (1994) [20]. To create a fully developed monolayer sheet, 100 µl/well (or 1 × 10^5^ cells/ml) was added to the 96-well tissue culture plate which was inoculated with 1 × 10^5^ cells/ml (100 µl/well) and incubated at 37°C for 24 h to develop a complete monolayer sheet. The growth media were decanted from the 96-well micro titer plate after the confluent sheet of cells was formed; the cell monolayer was washed twice with washing media. Two-fold dilutions of the tested samples were formed in RPMI media with 2% serum (maintenance medium). Briefly, 0.1 ml of each dilution was tested in different wells; three wells were designated as controls and received only maintenance medium. Plates were incubated at 37^°^C and evaluated. Cells were checked for any physical signs of toxicity, e.g., partial or complete loss of the monolayer, rounding, shrinkage, or cell granulation. The MTT (Bio Basic Canada, INC, Cat. No. T0793) solutions were prepared (5 mg/ml in PBS). Twenty µl of MTT solution were added to each well. Placed on a shaker at150 rpm for five min. and incubated at (37^°^C, 5% CO_2_) for 4 h to allow the MTT to be metabolized. The medium was discarded, and then the plates were dried using tissue paper to get rid of residues if required. Resuspend formazan in 200 µl dimethyl suphoxide. After that, place it on a shaker at 150 rpm for five minutes to mix the formazan into the solvent. Optical density was read using a microplate reader (BioTek ELx800 ELISA reader, Cat. No. 7351000, USA) at 560 nm, and the background was subtracted at 620 nm. Optical density ought to be directly correlated with cell quantity and compared to the doxorubicin as a control. The IC₅₀ values were calculated from dose–response curves using nonlinear regression analysis (GraphPad Prism v 9.0). The criteria for IC₅₀ were defined as the concentration required for reducing cell viability by 50% compared to untreated controls.

### Fatty acid methyl esters (FAME)

One gram of the ASSOE was transferred to a 5.0 ml lab vial, then 1.0 ml of methanol and chloroform (2:1, v/v) were added, and the mixture was agitated on the vortex mixer for three min. After that, the mixture was transferred to the Petri dish and allowed to dry at room temperature. Following drying, one ml of hexane and chloroform (1:1, v/v) was added to reconstitute, then treated with ten µl of N, O-Bis (trimethylsilyl) trifluoroacetamide using trimethyl-chlorosilane as the derivatization agent. The solution was then filtered by a 0.22 μm membrane filter after being agitated once more for 3 min. via the vortex. At last, one microliter of the filtrate sample solution was prepared for injection into the GC.

### Gas chromatography (GC) of ASSOE

Fatty acid methyl esters (FAME) were analyzed by a GC instrument (A HP 6890, Hamilton, CA, USA) equipped with an Innowax cross-linked polyethylene glycol column (30 m; i.d. 0.32 nm; 0.5 μm film width). Fatty acid methyl esters have been calculated as the appropriate fatty acid in the ASSOE using the procedure of (IUPAC- method 2.301 1987) [21]. Retention time of the fatty acids peak in the samples compared to the retention time of the standard was utilized to evaluate the fatty acid concentrations. Myristoleic, pentadecylic, palmitic, palmitoleic, stearic, oleic, linoleic, linolenic, and arachidic fatty acids were used as standards.

### Experimental animals and design

Forty-nine male Wistar albino rats weighing 180–200 g were obtained from the animal house of the National Center for Radiation Research and Technology, Egyptian Atomic Energy Authority, Cairo, Egypt. The rats were kept in standard conditions with regard to light, fresh air ventilation, room temperature (25^°^C), and humidity (60%). Recapture as the animals were allowed access to feed and water ad libitum the name the type of feed given. Also, the rats were assigned to seven groups of seven rats each (*n* = 7) as follows:

Group 1: Served as control and were administered normal saline orally.

Group 2: Rats subjected to γ-irradiation at 4 Gy whole body.

Group 3: Rats subjected to γ-irradiation at 6 Gy whole body.

Group 4: Rats subjected to γ-irradiation at 8 Gy whole body.

Group 5: 4 Gy γ-irradiated rats received an active dose of freshly made ASSOE at a dose of 200 mg/kg B.W. for 14 days following irradiation.

Group 6: 6 Gy γ-irradiated rats received an active dose of freshly made ASSOE at a dose of 200 mg/kg B.W. for 14 days following irradiation.

Group 7: 8 Gy γ-irradiated rats received an active dose of freshly made ASSOE at a dose of 200 mg/kg B.W. for 14 days following irradiation.

The oil extract was administered to the irradiated groups 5, 6, and 7 twenty-four hours after the irradiation treatment. The animal experiment was carried out at the National Center for Research Radiation and Technology (NCRRT), Egyptian Atomic Energy Authority, Cairo, Egypt, in accordance with the ARRIVE 2.0 guidelines [22] and under protocol serial number (F/6A/ 22) which was approved by the Research Ethics Committee of NCRRT. Ethical conduct adhered to the 3Rs principles (Replace, Reduce, and Refine) as outlined by CIOMS and ICLAS (2012).

### Irradiation procedure

The ^137^Cs gamma cell 40, which is placed in the NCRRT, was the source of the radiation. The cages with rats were put in the gamma cell’s chamber. Animals received doses of 4, 6, and 8 Gy at a dose rate of 0.175 Gy/min throughout the duration of the irradiation.

### Blood collection

After 14-day of treatment, animals were fasted for 10 h and subsequently anesthetized before sample collection. General anesthesia was induced via intraperitoneal (i.p.) injection of a ketamine–xylazine combination at a dose of 80 mg/kg ketamine and 10 mg/kg xylazine. The choice of ketamine–xylazine was based on its wide use and effectiveness in ensuring deep anesthesia for minor surgical and terminal procedures in rodents. After confirming a lack of response to paw pinch (ensuring adequate depth of anesthesia), euthanasia was performed via exsanguinations by cardiac puncture, followed by cervical dislocation to ensure death.

Whole blood was collected using sterile syringes into two tubes: one containing EDTA for hematological and immunological assessments (CD4, CD8, CRP, TNF-α), and the other left plain for serum separation and biochemical analysis. No animals were lost or excluded prior to euthanasia, and all procedures were performed by trained personnel to minimize animal discomfort and procedural variability.

### Immunological marker assessment

The CD4, CD8, CRP, and TNF-α concentrations were evaluated using enzyme-linked immunosorbent assay (ELISA) kits. CD4 and CD8 kits were sourced from MyBioSource (USA), while CRP and TNF-α kits were obtained from ALPCO Diagnostics (USA). All assays were performed according to the manufacturers’ protocols.

### Hematological analysis

Complete blood count (CBC) parameters, hemoglobin, red blood cells, white blood cells, hematocrit, as well as platelet calculation, were measured via an automated hematology analyzer (Swelab Alpha, Boule Diagnostics, Sweden).

### Biochemical assays

Total cholesterol and triglycerides were assayed utilizing an enzymatic colorimetric kit (BioMed Diagnostics, Egypt), following the guidelines provided by the manufacturer.

### Statistical analysis

The obtained results were presented as mean ± standard error (SE) and the statistical analysis was performed using two-way analysis of variance (ANOVA), and Duncan’s multiple range test for post hoc comparisons was applied to evaluate statistical significance difference at *p* < 0.05 [23]. The SPSS software version 25.0 (IBM, Armonk, NY, USA) is used to calculate the means and standard division, while the Costat is used to compare the means.

## Results and discussion

### Seed oil yield

*Annona squamosa* seed oil yield was 28.13 ± 2.58%, which gave a TPC of (0.716 ± 0.0200 mg/g oil) and TFC of (0.148 ± 0.010 mg/g) oil concentrations as indicated in Table 1. The steady free radicals used in the DPPH antioxidant behavior operate as radical scavengers by taking electrons from reactive radicals. The findings illustrated in Table 1 demonstrated the notable variance in the tested ASSOE scavenging capability against the DPPH radical percentages (65.41 ± 3.03%).

**Table 1:** Yield %, total phenol and total flavonoids contents, as well as antioxidant activity (DPPH %) of ASSOE

Sample	Yield (%)	Total phenolic content (mg/g oil)	Total flavonoids content (mg/g oil)	DPPH (%)
ASSOE	28.13 ± 2.58	0.716 ± 0.0200	0.148 ± 0.010	65.41 ± 3.03

ASSOE, *Annona squamosa* seed oil extract. Results are represented as means ± SE (*n* = 3)

Sharma et al. (2013) [24] showed that screening *A. squamosa* for phytochemicals may be utilized as a diagnostic method for standardizing therapeutic plants. The current data are in line with the latest results of Leite et al. (2021) [25], who showed that the extract from custard apple seed had much higher TPC and TFC values than the pulp. Polyphenols can scavenge free radicals directly or indirectly in their capacity as antioxidants. Because polyphenols stabilize reactive entities by giving free radicals electrons or hydrogen atoms, they have the capability of scavenging the free radicals [26]. Prior research by Arruda et al. (2018) [27] indicated a positive link between antioxidant capacity and phenolic compounds. The functional flavonoids’ hydroxyl groups functionally regulate their antioxidant effects via scavenging the free radical and/or chelate ions [28]. The biological activity of ASSOE is mostly caused by the presence of phenol compounds, alkaloids, peptides, amino acids, sterols, tannins, flavonoids, polysaccharides, and tocopherols [29]. Therefore, there is a great deal of promise for using the fruit’s inedible components (seeds) as natural antioxidant supplements. Additionally, they could be used to progress to create byproducts that can be used as functional components and nutraceuticals [30]. Although numerous research studies have found a correlation between plant phenolic content and antioxidant capacity, it was noted that data about the antioxidant potential of ASO are rare to find [31]. Various natural products, including flavonoids, phenylpropanoids, stilbenes, vitamin C, and active compounds from medicinal plants, play a significant role in ROS scavenging. By mitigating oxidative stress and reinforcing cellular defenses, these natural products hold promise as protective agents against the deleterious effects of ionizing radiation [7]. Moreover, recent studies have shown that polyphenols not only mitigate oxidative stress by directly neutralizing ROS generated by radiation but also establish a multi-layered radiation protection mechanism through the activation of DNA damage repair, inflammatory response, and cell death signaling pathways [32].

### Cytotoxicity of *Annona squamosa* seed oil

**Fig. 1:**
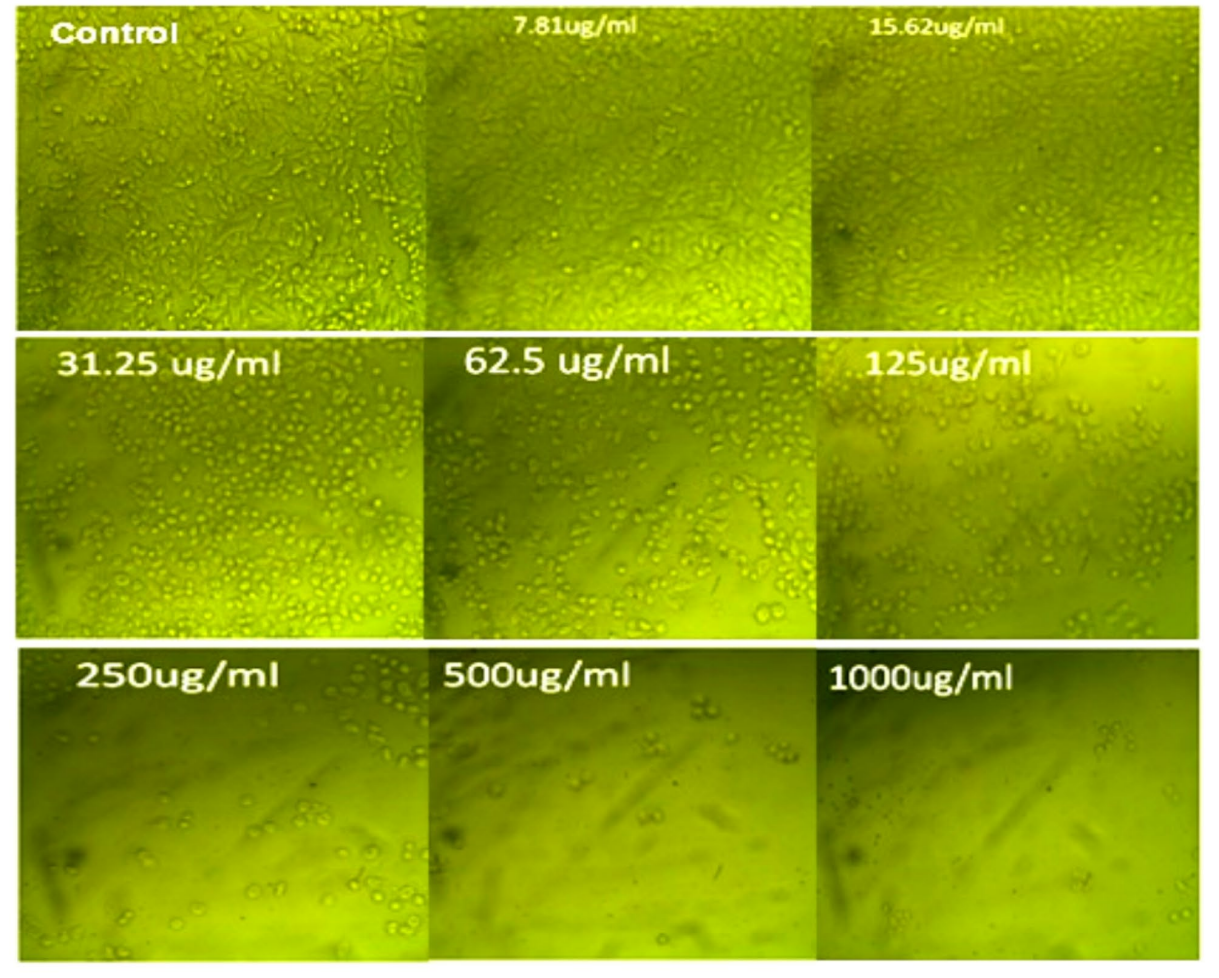
Effect of *A. squamosa* seed oil various concentrations 7.81, 15.62, 31.25, 62.5, 125, 250, 500, and 1000 µg/ml) on cancer cell line Caco-2. Data are presented as mean from three independent experiments performed in triplicate (*n* = 3). IC_50_ value was calculated (47.0 ± 0.68 µg/ml), and doxorubicin (1 µM) was used as the positive control

**Fig. 2:**
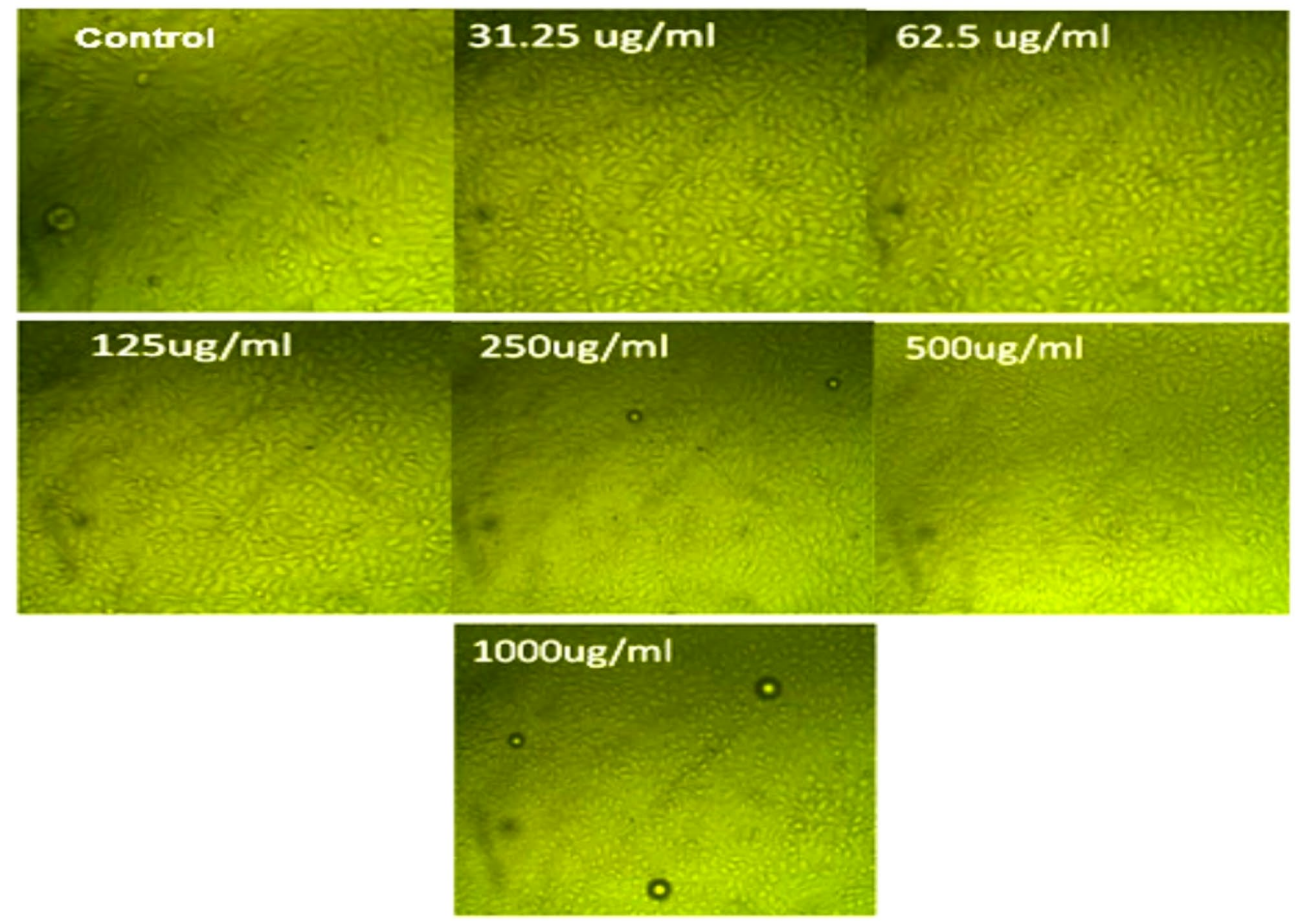
Effect of *A. squamosa* seed oil various concentrations (31.25, 62.5, 125, 250, 500, and 1000 µg/ml) on cancer cell line HepG-2. Data are presented as mean from three independent experiments performed in triplicate (*n* = 3). IC_50_ value was calculated (912.33 ± 10.81 µg/ml), and doxorubicin (1 µM) was used as the positive control

Findings obtained in Table 2; Figs. 1 and 2 exhibited an anti-cancer activity against the investigated cancer cell lines of Caco-2 (colorectal adenocarcinoma) and HepG-2 (hepatocellular carcinoma). The IC_50_ values of the ASSOE were 47 ± 0.68 µg/ml for Caco-2 and 912.33 ± 10.81 µg/ml for the HepG-2 cancer cell lines.

The secondary metabolites, including phenolic substances, have been shown to exhibit cytotoxic and cytostatic properties, suggesting that they may be classified as anti-tumor agents [33]. When *A. squamosa* L. leaf and seed extracts have been examined in vitro for their anticancer activities on MCF-7, it has been discovered that the maximal activity (IC_50_) of the seed extracts was superior to that of the leaf extracts.

Moreover, several substances such as vitamins, carotenoids, annonaceous acetogenins, alkaloids, tocols, and phytosterols, may also have an anticancer impact [34]. The ASSOE has demonstrated substantial antitumor activity in vitro and in vivo against human hepatoma cells, suggesting the potential for the extract to be developed as a novel anti-liver cancer medication [10].

Annona is a plant with excellent anticancer and cytotoxic potential to be used as an alternative or complementary therapy in various types of cancer because it has bioactive compounds such as acetogenins, phenolic compounds, flavonoids, and alkaloids, whose main effect is the apoptosis of cancer cells [35]. A novel family of substances known as anonaceous acetogenins has been shown to possess significant cell growth inhibitory properties. Three distinct mechanisms might account for *A. squamosa’s* anticancer activity: the first is the promotion of changed cells’ deadly differentiation, the second is its antiproliferative action, and the third is the modification of tumor cells’ surface antigens, which triggers the immune system [36]. Other findings proved that different ACG subtypes inhibited different types of cancer cells in different ways. The structure-activity connections of ACGs against various cancer cells, including several drug-resistant cancer cells, were investigated in vitro [37]. Additionally, Wang et al. (2014) [38] investigated the antitumor properties of the *A. squamosa* leaves’ aqueous and ethyl acetate extract against a variety of cancer cell lines, such as the human epidermoid carcinoma cell line KB-3-1 and the colon cancer cell line HCT-116, and indicated that the ethyl acetate extract was more effective than the aqueous extract. The current research suggests that the inedible fruit portions have potential use in pharmacology and biotechnology. Through oral administration, ASSO reduced the development of H22 tumor cells in mice by a maximum inhibitory rate of 53.54% [39].

**Table 2:** Effect of *A. squamosa* seed oil extract different concentrations (µg/ml) on Caco-2 and HepG-2 cancer cell lines

ID	Dose (µg/ml)	Mean O.D ± SE	Viability (%)	Toxicity (%)	IC_50_ ± SD (µg/ml)
Control	--------	0.779 ± 0.007638	100	0	
Caco-2	7.81	0.76967 ± 0.004096	99.18699	1.198117	47 ± 0.68
	15.62	0.772667 ± 0.005783	98.80188	0.813008	
	31.25	0.477333 ± 0.026773	61.27514	38.72486	
	62.5	0.270667 ± 0.007055	34.7454	65.2546	
	125	0.135000 ± 0.010693	17.32991	82.67009	
	250	0.084000 ± 0.002646	10.78306	89.21694	
	500	0.044667 ± 0.005608	5.733847	94.26615	
	1000	0.017000 ± 0.000577	2.182285	97.81772	
Control	--------	0.8680.0 ± 0709500	100	0	912.33 ± 10.81
HepG-2	31.25	0.860333 ± 0.006333	99.11674347	0.883256528	
	62.5	0.862000 ± 0.005196	99.30875576	0.69124424	
	125	0.862667 ± 0.004842	99.38556068	0.614439324	
	250	0.849333 ± 0.006566	97.84946237	2.150537634	
	500	0.821667 ± 0.002186	94.66205837	5.337941628	
	1000	0.364333 ± 0.003756	41.97388633	58.02611367	

Results are expressed as means ± SD (*n* = 3), from three independent experiments performed in triplicate (*n* = 3), Doxorubicin (1 µM) was used as the positive control. IC₅₀ (half-maximal inhibitory concentration) values were calculated using nonlinear regression analysis.

### Oil composition

The content of SFAs and USFAs is disclosed by the fatty acid composition, which is utilized as a health indicator to evaluate the oxidative stability of fat and oil [40]. The different fatty acids found in ASSOE were identified using gas chromatography (GC). Table 3 included nine different fatty acids. Oleic (57.80%) and linoleic (23.31%) were the two most predominant fatty acids in the ASSOE, along with palmitic acid (16.22%). Analysis furthermore revealed that the oil included minor quantities of arachidic (1.27%), linolenic (0.93%), stearic (0.21%), palmitoleic (0.17%), pentadecylic (0.05%), and myristoleic acids (0.04%). The ASSOE revealed a significant concentration of UFAs, of 82.25%, contributed by oleic acid (57.80%), linoleic acid (23.31%), myristoleic (0.04), palmitoleic acid (0.17), and linolenic acid (0.93%). Additionally, it was discovered that the oil included the SFAs: pentadecylic acid (0.05%), stearic acid (0.21%), arachidic acid (1.27%), and palmitic acid (16.22%), which collectively contributed about 17.75% of the oil. The fatty acid profile utilized as a healthiness indicator towards evaluating the oxidative constancy of fats and oils reveals the total content of SFAs as well as USFAs [13]. They provided that the following percentages of palmitic, stearic, oleic, and linoleic fatty acids were found in ASSOE in that arrangement of 15.6%, 10.6%, 49.2%, and 22.3%, respectively.

**Table 3:** Evaluation of fatty acid Methyl esters of ASSOE using GC

Peak No.	Fatty acid	No. of carbon atoms	Relative percentage (%)
1	Myristoleic acid	C14:1	0.04
2	Pentadecylic acid	C15:0	0.05
3	Palmitic acid	C16:0	16.22
4	Palmitoleic acid	C16:1	0.17
5	Stearic acid	C18:0	0.21
6	Oleic acid	C18:1	57.80
7	Linoleic acid	C18:2	23.31
8	Linolenic acid	C18:3	0.93
9	Arachidic acid	C20:0	1.27
UFAs	82.25	-	-
SFAs	17.75	-	-
Total FAs	100	-	-

UFAs: Unsaturated fatty acids, SFAs: Saturated fatty acids, FAs: Fatty acids

Fatty acids (FAs) are vital energy-storage compounds and primary components of plant membrane lipids; their composition modulates membrane properties like fluidity. The FA composition of lipids in mesocarp tissues has a crucial role during the ripening and senescence processes of the CA fruit. Palmitic acid (C16:0), stearic acid (C18:0), oleic acid (C18:1), and linoleic acid (C18:2) were the major FAs in the edible parts, and the highest level of FAs, especially unsaturated ones, was found in custard apple seed [41]. The ASSOE contain varying proportions of SFAs and UFAs tied in their TAG (triacylglyceride) molecules, and the percentage of UFAs is higher than that of SFAs (i.e., FAs esterified to a glycerol moiety). Additionally, this makes ASSOE an interesting source of two PUFAs, linoleic and linolenic acids, which are termed EFAs (essential fatty acids) because humans must obtain them from their diets [11]. The observed protective effects can be attributed to the diverse bioactive profile of ASSOE. Its richness in oleic acid may play an important role in dampening radiation-induced inflammatory cascades, since oleic acid has been shown to suppress NF-κB and MAPK signaling and reduce pro-inflammatory cytokine production [42]. Linoleic acid contributes to oxidative defense by reducing ROS levels and enhancing glutathione synthesis, restoring redox homeostasis under stress conditions [43]. Furthermore, tocopherols and phenolic compounds found in ASSOE act as potent free radical scavengers, protecting cellular macromolecules from oxidative injury and lipid peroxidation [25]. Beyond these antioxidant components, annonaceous acetogenins, characteristic of Annona species, exert cytotoxic and immunomodulatory effects through mitochondrial complex I inhibition and induction of apoptosis, mechanisms that may contribute to the modulation of immune cell profiles in irradiated animals [44]. Similarly, *Nigella sativa* seed oil and its active component thymoquinone have shown consistent radioprotective activity in animal models by enhancing antioxidant defenses and reducing oxidative injury in multiple organs [45, 46].

The current results corroborate earlier reports that demonstrated the GC-MS analyses of ASSOE contained a mix of 71.28% USFAs (C16 to C21) and 24.17% saturated fatty acids, with their methyl esters, which are mainly made up of two PUFAs: 18:1 oleic (33.5%) and 18:2 linoleic (16%) [47].

A significant part of the whole fatty acids under study (75.55%) are USFAs, which are abundant in seed oil. Nonetheless, 95.22% of the USFAs are made up of oleic and linoleic acids. *Annona* seed oil can lower blood serum cholesterol because it contains higher USFAs than SFAs, which are more edible as food [48]. The transformation of linoleic to γ-linolenic and arachidonic acid preserves the stratum corneum’s permeability and suppresses pro-inflammatory cytokines and eicosanoids [49]. Based on the aforementioned findings, it is possible to conclude that oils obtained from *A. squamosa* have a higher proportion of USFAs than saturated ones, making them a potential plant oil that might be a source of nutritive edible oil. The ASSOE contains a significant amount of tocopherols and is a substantial source of USFAs, namely oleic and linoleic acids.

### Immunological markers

As shown in Table 4, after 7 days of gamma irradiation, the serum contents of CD4 and CD8 (pg/ml) decreased significantly from 135.20 to 325.74 to 78.11 and 241.96 in group 2; to 68.32 and 201.33 in group 3; and to 56.25 and 152.62 pg/ml in group 4, respectively, in comparison to reference animals in group 1. Comparing the effective dose of ASSOE to the equivalent irradiation groups 2, 3, and 4, the concentrations of CD4 and CD8 (pg/ml) were considerably increased to 110.74 and 301.51 in group 5, 85.47 and 396.22 in group 6, and 93.12 and 244.94 pg/ml in group 7.

Table 4 shows that after a 14-day therapy with ASSOE, there was a considerable increase in CD4 and CD8 contents. CD4 concentrations rose to 129.71, 123.50, and 116.47 ng/ml in groups 5, 6, and 7 as compared to their respective irradiated groups 2, 3, and 4. In groups 5, 6, and 7 CD8 levels rose to 326.44, 312.95, and 287.50 pg/ml, respectively, in comparison to equivalent irradiated groups 2, 3, and 4. The blood cytokine levels of TNF-α and CRP in rats subjected to radiation are shown in Table 4. After 7 days of irradiation, serum CRP values in groups 2, 3, and 4 were found to be considerably higher than those in group 1 of control rats, at 3.85, 7.52, and 8.63 ng/ml, respectively. After 14 days of radiation, the CRP levels in the same groups reduced marginally to 3.22, 5.39, and 7.02 ng/ml. The increased levels of CRP were considerably reduced to 1.80, 4.65, and 4.01 ng/ml in groups 5 and 6 after 7 days and to 1.09, 3.27, and 3.11 ng/ml for the same groups after 14 days, following the administration of the active dose of ASSOE. Following irradiation, serum TNF-α concentration in groups 2, 3, and 4 increased to 36.54, 72.41, and 95.11 pg/ml after 7 days, respectively, in comparison to group 1 (control rats), as shown in Table 4. After seven days of therapy, the administration of ASSOE dramatically decreased the high levels of TNF-α to 27.47, 35.60, and 32.95 pg/ml for the identical groups. Additionally, compared to the comparable groups, rats treated with oil extract for 14 days showed greater recovery, with TNF-α concentrations dropping to 15.37, 12.78, and 21.62 pg/ml for groups 5, 6, and 7, respectively (2, 3, and 4). *A. squamosa* oil extract was chosen for this study as a potential remedy for the immunesuppressive impacts of gamma rays, as it contains several bioactive components, like flavonoids and phenols, which scavenge reactive oxygen species generated in the body and have antioxidant properties. The function of T cells in cell-mediated immunity is well established, while B cells are mostly in charge of humoral immunity by producing antigen-specific antibodies that neutralize extracellular microbial toxins [49].

In general, CD4 and CD8 are utilized to identify cytotoxic and helper T cells, respectively. When major histocompatibility complex (MHC) class II-bound peptide antigens are presented to CD4 + cells, they become activated. They proliferate speedily and release tiny proteins known as cytokines, which aid in or control the immune response. As a result, their up- or down-regulation has significant implications in a variety of disease circumstances. However, the majority of cytotoxic T lymphocytes have receptors that are able to identify peptides attached to class I MHC molecules.

Radiation-induced immune system motivation or depression can result in impaired immunological function. By reacting to outside stimuli substances, the immune system is in charge of preserving an organism’s integrity. In the current investigation, peripheral blood CD4 contents significantly decreased at the used gamma rays dose levels (4, 6, and 8 Gy), while CD8 contents also reduced concurrently. Due to their ability to scavenge ROS, ASSOE treatment for 14 days raises blood CD4 and CD8 concentrations in irradiated groups. Several radioprotective agents mitigate radiation injury by accelerating cell repair, including hormones or steroid analogs, such as estrogens and androgens and interleukins, TNF, hematopoietic growth factors, interferons, and immunomodulatory peptides. These agents primarily function by activating the NF-kB pathway. The NF-kB is a rapid-acting primary transcription factor that is inactive in the cytoplasm. It can be activated by IL-1b, TNF, lipopolysaccharide receptors, bacterial and viral antigens, ROS, and ionizing radiation [50].

**Table 4:** Effect of ASSOE treatment (200 µg/kg B.W.) for 7 and 14 days on serum concentrations of CD4, CD8 (pg/ml), TNF (pg/ml), and CRP (ng/ml) of γ-irradiated male albino rats

Treatments	Parameter							
	CD4		CD8		CRP		TNF-α	
	7 days	14 days	7 days	14 days	7 days	14 days	7 days	14 days
Control	135.20 ± 2.790^a^	137.36 ± 2.67^a^	325.74 ± 3.83^b^	336.57 ± 4.69^a^	1.05 ± 0.12^f^	1.13 ± 0.23^f^	18.31 ± 1.17^i^	19.52 ± 0.74^hi^
4 Gy	78.11 ± 2.44^j^	120.25 ± 76^cd^	241.96 ± 1.88^g^	252.19 ± 2.72^f^	3.85 ± 0.32^de^	3.22 ± 0.42^e^	36.54 ± 2.04^de^	36.41 ± 1.43^de^
6 Gy	68.32 ± 3263^k^	112.25 ± 2.01^ef^	201.33 ± 2.31^i^	227.20 ± 2.71^h^	7.52 ± 0.38^ab^	5.39 ± 0.58^c^	72.41 ± 2.97^b^	39.22 ± 1.09^d^
8 Gy	56.25 ± 2.40^l^	102.48 ± 1.85^g^	152.62 ± 2.13^k^	188.12 ± 2.74^j^	8.63 ± 0.69^a^	7.02 ± 0.46^b^	95.11 ± 3.45^a^	53.12 ± 1.87^c^
Oil + 4 Gy	110.74 ± 2.01^f^	129.71 ± 2.72^b^	301.51 ± 2.69^c^	326.44 ± 3.89^e^	1.80 ± 0.13^f^	1.09 ± 0.13^f^	27.47 ± 0.48^g^	15.37 ± 0.89^j^
Oil + 6 Gy	85.47 ± 2.90^i^	123.50 ± 2.95^c^	296.22 ± 2.72^d^	312.95 ± 4.13^c^	4.65 ± 0.67^cd^	3.27 ± 0.56^e^	35.60 ± 1.87^ef^	12.78 ± 0.95^j^
Oil + 8 Gy	93.12 ± 2.12^h^	116.±3.77^de^	244.94 ± 2.67^g^	287.50 ± 2.91^e^	4.01 ± 0.84^de^	3.11 ± 0.54^e^	32.95 ± 1.72^f^	21.62 ± 0.65^h^

Cluster of Differentiation4 (CD4), Cluster of Differentiation8 (CD8), C-Reactive Protein (CRP) and Tumor Necrosis Factor-α (TNF-α). The provided data are means ± standard errors (SE) (*n* = 5). At *p* < 0.05, different superscript letters point to a significant difference

CRP is a general indicator of inflammation. The detection of CRP in plasma is a simple sign that the body has begun to activate its non-specific defenses [51]. Cancer patients who get radiation treatment had higher CRP levels [52]. *Vernonia cinerea* extract reduced CRP and TNF-α levels in radiation-exposed mice, indicating its anti-inflammatory effects [53]. These two pro-inflammatory indicators were lower in groups treated with a blend of green tea and grape seed extract (1:2) than in those that were not exposed to radiation, suggesting that the mixture had anti-inflammatory properties [54]. The TNF-α is considered to have a significant role in the pathogenesis of irradiation pneumonia. TNF-α induces inflammation by preparing leukocytes to synthesize oxidants, triggering prostaglandin and other inflammatory mediators, and causing adhesion molecules to be produced, attracting leukocytes to tissue injury sites [55]. The three main causes of radiation-induced damage are inflammation, deoxyribonucleic acid (DNA) damage, and oxidative stress [56]. According to the current study, a number of studies have consistently revealed that radiation-induced activation and expression of the genotoxic stress-induced pathways occur after oxidative stress.

Tumor Necrosis Factor-α and other cytokines are produced in greater amounts as a result of this inflammatory condition [57]. Through their ability to further activate both immunological and non-immune cells, cytokines are essential for immune function and repair processes [58]. According to research conducted in vivo, a 10 Gy whole-body irradiation caused a series of inflammatory effects that included elevated TNF-α and IL-6 levels [59]. The blood levels of CRP and TNF-α cytokines were increased in rats given whole-body gamma irradiation. These two CRP and TNF-α cytokine indicators were decreased in groups treated with ASSOE for 14 days compared to those that were exposed to radiation, suggesting that the oil extract had anti-inflammatory properties. A previous study concluded that low concentrations of free curcumin protected cells from radiation via increased scavenging of free radicals, activation of the Nrf2 pathway (thus leading to increased expression of total antioxidant and thiol levels), and upregulation of antioxidant gene expression [60].

### Hematological indices

Rats irradiated with gamma radiation exhibited a noteworthy decline in platelet count, Hb concentration, RBC count, WBC count, and HCT percentage in comparison to control rats (Table 5). Treatment with ASSOE at (200 mg/kg B.W.) (groups 5, 6, and 7) led to a substantial (*p* < 0.05) enhancement in red blood cells, white blood cells, platelet count, hemoglobin content, and hematocritpercentage comparing to γ-irradiated groups (2, 3, and 4). These improvements were close to the control values. Hemoglobin concentration of groups 2, 3, and 4 was greatly reduced to 10.15, 9.76, and 8.52 g/dl, respectively, after 7 days from γ-irradiation compared with the control group (12.91 g/dl), but after 14 days from γ-irradiation, Hb concentration decreased to 10.93, 10.28, and 9.02 g/dl, respectively for the same groups compared with control group (13.31 g/dl). The Hb concentrations for groups 5, 6, and 7 increased significantly after receiving the active dose of ASSOE at dose of 200 mg/kg B.W.; after 7 days of treatment, they were 10.93, 9.94, and 9.11 g/dl, respectively, and after 14 days, they were 11.84, 10.97, and 10.41 g/dl, respectively, in comparison to groups 2, 3, and 4.

Red blood cell (RBC) count was decreased after γ-irradiation to 4.97, 3.15, and 2.23 (×10^12^/l), respectively after 7 days from γ-irradiation and to 5.11, 3.69, and 2.74 (×10^12^/l), respectively after 14 days from γ-irradiation for groups 2, 3, and 4 in comparison with control group 6.54 and 6.67(×10^12^/l), respectively.

**Table 5:** Effect of ASSOE treatment (200 mg/kg B.W.) for 7 and 14 days on hematological parameters of γ-irradiated male albino rats

Treatments	Parameters									
	Hb Conc. (g/dl)		RBC’s (× 10^12^/l)		HCT (%)		Platelets (×10^9^/l)		WBC’s (×10^9^/l)	
	7 days	14 days	7 days	14 days	7 days	14 days	7 days	14 days	7 days	14 days
Control	12.91 ± 1.92^a^	13.31 ± 1.24^a^	6.54 ± 0.91^a^	6.67 ± 0.94^a^	38.15 ± 6.46^a^	37.16 ± 1.83^ab^	443.14 ± 9.38^a^	445.11 ± 9.58^a^	7.68 ± 1.04^ab^	7.81 ± 0.90^a^
4 Gy	10.15 ± 1.89^cd^	10.93 ± 1.25^bc^	4.97 ± 0.66^bc^	5.11 ± 0.84^bc^	34.13 ± 2.41^cde^	33.92 ± 1.57^de^	296.51 ± 8.75^e^	311.14 ± 7.08^d^	5.51 ± 0.89^cdef^	5.83 ± 0.57^cde^
6 Gy	9.76 ± 1.25^ef^	10.28 ± 1.32^cd^	3.15 ± 0.43^ef^	3.69 ± 0.49^def^	30.64 ± 2.20^fg^	31.05 ± 1.39^fg^	231.14 ± 6.24^g^	259.38 ± 2.80^f^	4.03 ± 0.61^ghi^	4.23 ± 0.350^fghi^
8 Gy	8.52 ± 1.05^ef^	9.02 ± 1.34^def^	2.23 ± 0.54^g^	2.74 ± 0.43^fg^	26.42 ± 1.16^i^	27.13 ± 1.10^hi^	171.19 ± 3.32^j^	203.49 ± 3.70^i^	2.81 ± 0.23^i^	3.11 ± 0.29^hi^
4 Gy + oil	10.93 ± 1.45^bc^	11.84 ± 1.11^ab^	5.38 ± 0.48^a^	5.91 ± 0.86^ab^	35.61 ± 7.59^bcd^	36.21 ± 1.07^abc^	315.26 ± 9.58^d^	389.14 ± 7.08^b^	6.11 ± 0.93^bcd^	6.92 ± 0.82^abc^
6 Gy + oil	9.94 ± 1.90^cde^	10.97 ± 0.83^bc^	3.87 ± 0.28^de^	4.92 ± 0.77^bc^	32.54 ± 2.02^ef^	33.71 ± 1.62^de^	298.70 ± 7.79^e^	340.12 ± 6.91^c^	5.13 ± 0.40^defg^	6.11 ± 0.98^bcd^
8 Gy + oil	9.11 ± 1.85^7^	10.41 ± 0.95^bcd^	2.91 ± 0.14^efg^	3.88 ± 0.29^de^	28.94 ± 2.60^gh^	30.25 ± 2.09^g^	220.41 ± 13.49^h^	314.55 ± 12.57^d^	3.90 ± 0.35^ghi^	4.56 ± 0.74^efgh^

Data are means (*n* = 5) ± standard errors (SE). Distinct superscript letters at *p* < 0.05 show a significant difference

After treatment with ASSOE, RBC counts elevated to 5.38, 3.87, and 2.90 (×10^12^/l) after 7 days and to 5.91, 4.92, and 3.88 (×10^12^/l) after 14 days of treatment for groups 5, 6, and 7, respectively. Hematocrit percentage and platelet count were decreased after 7 and 14 days by increasing the dose level of γ-irradiation (4, 6, and 8 Gy) and then increased by treatment with ASSOE for 14 days. WBC count is similarly lower in groups 2, 3, and 4 than in the control groups at 7 and 14 days post-irradiation, falling to 7.68, 5.51, and 4.03 (×10^9^/l) and 5.83, 4.23, and 3.11 (×10^9^/l), respectively. Following treatment with ASSOE, it rose to 6.11, 5.13, and 3.96 (×10^9^/l) in groups 5 and 6 and 6.92, 6.11, and 4.56 (×10^9^/l) in groups 7 and 5, respectively.

In this study, the RBCs, WBCs, platelet count, Hb, and HCT % all significantly decreased after receiving doses of 4, 6, and 8 Gy of whole-body gamma irradiation. According to Abd El-Hady et al. (2024) [61], on the fourteenth day after the irradiation procedure, rats treated to fractionated doses of whole-body gamma irradiation (2 Gy, 4 times, every 3 days) up to 8 Gy showed substantial decreases in hemoglobin, red blood cells, MCH, MCHC, white blood cell count, and platelet count. Additionally, the HCT percent and MCV significantly decreased in comparison to the control values. Related findings published by Elshater et al. (2014) [62] revealed that rats given a single radiation treatment (5 Gy) saw a considerable drop in red blood cells, white blood cells, platelets, hemoglobin, and hematocrit percent. There was a noticeable difference in WBC and RBC counts in the bone marrow of cancer patients who had radiation therapy because radiation can cause bone marrow syndrome and harm hematopoietic stem cells [63]. Moreover, Zhong et al. (2017) [64] stated that after exposing mice to 6 Gy of radiation, they saw notable reductions in red blood cells, platelets, white blood cells, and lymphocytes in the peripheral blood cell count. Akomolafe and Chetty (2021) [65] revealed that the given radiation doses significantly lowered the number of neutrophils in the experimental animals, as the neutrophils are the most crucial protector present in the WBC that fights against infection. Additionally, Akinade and Akomolafe (2023) [66] cited that, the possible mechanism of radioprotectors of plant origin that have been identified includes scavenging free radicals, stimulating hematopoiesis, activating DNA repairing enzymes, reducing apoptosis, and boosting the immune system.

Abojassim et al. (2015) [67] used 6 Gy radiation on albino mice to investigate liver function, hematological system damage, and histological alterations in liver tissues. Hematocrit, mean corpuscular volume (MCV), mean corpuscular hemoglobin (MCH), RBCs, and Hb levels were all lower in the group subjected to 6 Gy of X-rays. This reduction in RBCs led to a corresponding drop in hemoglobin level and a decline in hematocrit % (Table 5). Alternatively, groups of irradiated animals treated with ASSOE showed important enhancements of RBCs, WBCs, platelets, Hb, and HCT percentages. The current results support the findings of Akomolafe and Chetty (2022) [68], who reported that exposure of mice to 4 and 8 Gy of X-ray radiation significantly reduced leukocyte counts. The reduction in leukocyte levels recorded among the irradiated groups may be attributed to the loss of lymphocytes. The lymphocytes are responsible for fighting infection and helping build the body’s immune system. A significant reduction in lymphocyte counts could lead to a lymphocytopenia condition.

In recent years, natural products have attracted growing interest from researchers in drug development, owing to their benefits, including multi-target synergistic effects, low biological toxicity, and the vast diversity found in natural compound libraries [69].

### Biochemical analysis

Table 6 shows that after 14 days of gamma irradiation, the blood levels of cholesterol were considerably higher in the γ-irradiated groups (2, 3, and 4) than in the control group, reaching 135.14, 148.57, and 158.24 mg/dl for cholesterol, respectively. In comparison to the control group (group 1), triglyceride levels rose to 202.29, 244.30, and 271.38 mg/dl, respectively after 14 days of gamma radiation. The elevated cholesterol levels in the -irradiated rats were reduced to 120.43, 124.30, and 139.56 mg/dl and the triglyceride concentrations were reduced to 139.20, 152.20, and 162.22 mg/dl in the groups that received the active dose of ASSOE for 14 days (5, 6 and 7).

**Table 6:** Impact of ASSOE treatment (200 mg/kg B.W.) for 7 and 14 days on levels of triglycerides and cholesterol in rats exposed to gamma radiation

Treatments	Parameters			
	Cholesterol (mg/dl)		Triglycerides (mg/dl)	
	7 days	14 days	7 days	14 days
Control	101.32 ± 2.71^i^	109.86 ± 2.71^g^	108.31 ± 3.17^l^	132.19 ± 2.67^j^
4 Gy	112.38 ± 2.08^g^	135.14 ± 2.68^c^	175.27 ± 1.21^f^	202.29 ± 2.98^d^
6 Gy	129.22 ± 1.80^d^	148.57 ± 2.36^b^	201.33 ± 3.62^d^	244.38 ± 2.77^b^
8 Gy	138.25 ± 2.79^c^	158.24 ± 3.05^a^	221.74 ± 2.55^c^	271.47 ± 2.68^a^
4 Gy + oil	98.24 ± 2.80^i^	120.43 ± 2.66^f^	124.05 ± 1.05^k^	139.20 ± 2.38^i^
6 Gy + oil	105.11 ± 2.04^h^	124.30 ± 2.78^e^	176.58 ± 1.23^f^	152.20 ± 2.27^h^
8 Gy + oil	121.57 ± 2.10^ef^	139.56 ± 2.66^c^	184.37 ± 1.17^e^	162.22 ± 2.49^g^

The data are means ± standard errors (*n* = 7). Different superscript letters pointed to significantly differed at *p* < 0.05

The latest findings showed that the levels of triglycerides and cholesterol had changed. These changes may have been caused by ionizing radiation, which causes harmful biological effects by radiolyzing water, which causes cells to create ROS. An inflammatory response can be triggered by ROS [70]. The overproduction of ROS has previously been linked to radiation harming biological tissues [71]. Proteins, lipids, carbohydrates, and nucleic acids are examples of biological macromolecules harmed by oxidative stress, which is made worse by oxidant/antioxidant disturbances. An excess of ROS exacerbates lipid peroxidation in live cells. In addition to producing more reactive oxygen molecules and upsetting cellular equilibrium, this initiates more oxidative damage [72]. These findings of Elmas et al. (2022) [2] suggested that *Annona muricata* has a radioprotective effect, possibly owing to its antioxidative and anti-inflammatory effects. Treatments using *Annona muricata* extracts seem to be promising as methods of radioprotection in patients receiving radiotherapy; however, much research is required to identify the mechanisms of these effects and their value in humans.

The current findings align with those of Abd El-Hady et al. (2024) [61], who discovered that fractionated doses of γ-radiation significantly raised the blood levels of HDL, LDL, triglycerides, and cholesterol compared to the control rats. Rats given both quercetin and curcumin before radiation treatment showed a considerable drop in cholesterol, with a nonsignificant drop in LDL levels and a greatly significant drop in both TG and HDL levels when compared to the values of the irradiated group. Kaleem et al. (2006) [73] used *A. squamosa* extract (ASE) on diabetic rats and saw improvements in lipemia, glycemia, and levels of antioxidant enzymes, indicating that it may be useful in reducing lipid peroxidation and other early and long-term problems from diabetes. Gupta et al. (2008) [74] assessed the effects of ASE and found that it dramatically reduced total cholesterol and triglycerides while increasing the activity of antioxidant enzymes in several tissues.

Due to damage to the cell membrane caused by gamma radiation, enzymes were released into the bloodstream. A decrease in HDL levels and an increase in total triglycerides, total cholesterol, LDL, and VLDL contents in serum might be caused by lipids being released into the bloodstream through damaged cell membranes [75].

However, LDL, HDL, and the LDL/HDL ratios were significantly elevated in response to γ-radiation. This suggests that γ-radiation caused tissues to undergo apoptosis and lipid peroxidation. Irradiation generates free radical producing oxidative stress, which altered lipid metabolism and can be a major reason of hormonal imbalance. This imbalance induces hyperlipidemia, hypercholesterolemia and hypertriglyceridemia. The hyperlipidemic status appeared post irradiation assign to the stimulation of liver enzymes and biosynthesis of fatty acids, the mobilization of fats from the adipose tissues to the blood stream and mitochondrial dysfunction [76].

According to these findings, the ASSOE contains greater concentrations of linoleic and linolenic acids, which may be the cause of the reduction in triglyceride and cholesterol levels. As an omega-6 fatty acid, linoleic acid is utilized in the manufacture of arachidonic acid and, therefore, a number of prostaglandins, thromboxane, and leukotrienes that are referred to as eicosanoids. Because it lowers LDL cholesterol and lowers the danger of heart diseases, linoleic acid is vital for keeping heart health [77]. Linoleic and linolenic acids are two distinct vital fatty acids that are often present in plants but are not generated by humans [78]. They are promoted as health supplements because of their linked health advantages, which include the potential to prevent and treat coronary heart diseases and several chronic illnesses [79].

## Conclusion

According to the current study, ASSOE may offer protection against the immunosuppressive effects of gamma irradiation as well as its biochemical and hematological alterations. Reducing the amount of ROS created by radiation, raising the levels of CD4 and CD8, lowering the TNF-α and CRP contents, and raising the hematological components in the peripheral blood. After 14 days of therapy, biochemical markers such as triglycerides and cholesterol also dropped. The oil extract from *A. squamosa* may be utilized as a radioprotector and is a promising option for preventing the immunosuppressive effects of gamma radiation.

ASSOE was found to be effective in key bioactivities, including antioxidant activity and antitumor/anticancer activity, and based on in vivo and in vitro investigations, secondary metabolites of ASSOE cause anticancer and other immune system-associated effects. Hence, to isolate and identify the active metabolites that contribute to their robust anti-inflammatory and anticancer actions, future research on ASSOEs should concentrate on broad phytochemical investigations. A few studies were available on the phytochemical profile and the molecular mechanism of various bioactivities of *A. squamosa* seeds. However, more pharmacological studies need to be performed to determine the nutraceutical and food supplement potential of the seeds. Based on the studies available, *A. squamosa* seeds may likely be used as an ingredient in the nutraceutical and food/nutrition industry, especially as anticancer drugs and antitumoral dietary supplements, benefiting human health.

## Data Availability

The data sets used and/or analyzed during the current study available from the corresponding author on reasonable request.
